# Targeting Endothelial Dysfunction in Vascular Complications Associated with Diabetes

**DOI:** 10.1155/2012/750126

**Published:** 2011-10-13

**Authors:** Arpeeta Sharma, Pascal N. Bernatchez, Judy B. de Haan

**Affiliations:** ^1^Oxidative Stress Laboratory, Diabetic Complications Division, Baker IDI Heart and Diabetes Institute, P.O. Box 6492, St. Kilda Road Central, Melbourne, VIC 8008, Australia; ^2^The James Hogg Research Centre at St. Paul's Hospital and Department of Anesthesiology, Pharmacology and Therapeutics, University of British Columbia, Vancouver, BC, Canada V6Z 1Y6

## Abstract

Cardiovascular complications associated with diabetes remain a significant health issue in westernized societies. Overwhelming evidence from clinical and laboratory investigations have demonstrated that these cardiovascular complications are initiated by a dysfunctional vascular endothelium. Indeed, endothelial dysfunction is one of the key events that occur during diabetes, leading to the acceleration of cardiovascular mortality and morbidity. In a diabetic milieu, endothelial dysfunction occurs as a result of attenuated production of endothelial derived nitric oxide (EDNO) and augmented levels of reactive oxygen species (ROS). Thus, in this review, we discuss novel therapeutic targets that either upregulate EDNO production or increase antioxidant enzyme capacity in an effort to limit oxidative stress and restore endothelial function. In particular, endogenous signaling molecules that positively modulate EDNO synthesis and mimetics of endogenous antioxidant enzymes will be highlighted. Consequently, manipulation of these unique targets, either alone or in combination, may represent a novel strategy to confer vascular protection, with the ultimate goal of improved outcomes for diabetes-associated vascular complications.

## 1. Introduction

Diabetes mellitus is a highly prevalent chronic metabolic disorder that is considered a major health problem in westernized societies [1–3]. Diabetes, characterized by persistent elevation of blood glucose levels (hyperglycaemia), occurs due to inadequate production of insulin (type 1 diabetes; T1D), or resistance to endogenous insulin usually associated with the metabolic syndrome and obesity (type 2 diabetes; T2D). Despite intensive glycaemic control, individuals with T1D and T2D are predisposed to developing vascular complications, which include cardiomyopathy, atherosclerosis, nephropathy, retinopathy, and neuropathy [4, 5]. Indeed, there is a striking correlation between the incidence of cardiovascular disease and mortality rates in diabetic patients [6, 7]. Although the mechanisms by which diabetes increases cardiovascular complications are incompletely understood, strong supportive evidence from experimental and clinical studies points to the impaired function of the vascular endothelium as a critical inducer of these cardiovascular complications [4, 8]. In the current review, we discuss the important role of the endothelium and the factors that contribute to diabetes-associated cardiovascular complications, in particular nitric oxide (NO) bioavailability and reactive oxygen species (ROS) generation. In addition, this review will highlight novel compounds or molecules that show promise in improving vascular function in diabetic settings. 

## 2. The Protective Nature of the Vascular Endothelium

**Figure 1:**
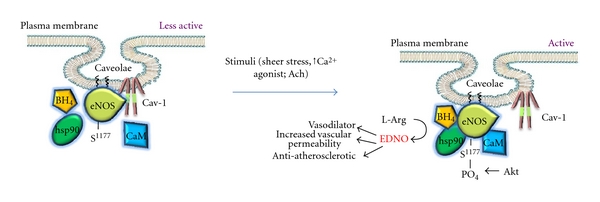
Under basal conditions, eNOS is kept in a “less active” state by binding to Cav-1. Activation of eNOS occurs by stimuli, (sheer stress, agonists such as acetylcholine, Ach), which causes an increase in intracellular Ca^2+^ and subsequent recruitment of cofactors and phosphorylation of the enzyme at S^1177^. Following which, eNOS is dissociated from Cav-1 and efficient EDNO production occurs. CaM: calmodulin; hsp90: heat shock protein 90.

The vascular endothelium, comprised of a single layer of endothelial cells that line the lumen, was initially only considered as a physical barrier separating the circulating blood from the underlying tissue. However, over the past few decades, the versatile role of the endothelium in cardiovascular homeostasis has become more greatly appreciated. The vascular endothelium is responsible for maintaining vascular tone and blood pressure which is achieved by balancing the release of vasoconstrictors and vasodilators [9]. In addition, the vascular endothelium maintains blood fluidity by promoting anticoagulant, antiatherosclerotic and antithrombotic pathways [9]. Endothelial-derived nitric oxide (EDNO), a potent gaseous mediator released by endothelial cells, is widely accepted as the key determinant of endothelial function. Importantly, EDNO directly induces vascular smooth muscle relaxation by the activation of soluble guanylate cyclase and subsequent increase in cGMP [10], thereby contributing to resting vascular tone and blood pressure. EDNO is also considered an anti-atherogenic and antithrombotic molecule through its ability to inhibit platelet aggregation, inflammatory cell adhesion, and smooth muscle cell proliferation and migration [11]. Constitutively expressed endothelial nitric oxide synthase (eNOS) converts L-arginine to nitric oxide using molecular oxygen in the presence of its cofactors tetrahydrobiopterin (BH_4_), heat shock protein 90 (hsp90), and calcium-calmodulin complex (Figure 1). Additionally, eNOS activity is significantly increased upon phosphorylation at serine 1177, mediated by the protein kinase B/Akt pathway [12]. Importantly, eNOS is only catalytically active upon dissociation from its endogenous binding protein, caveolin-1 (Cav-1) [13], which maintains eNOS in a tonic “inhibitory” state and will be discussed in greater detail below. Indeed, functional alterations in EDNO production or bioavailability play a critical role in the pathogenesis of various cardiovascular diseases [14]. Therefore, regulation and/or improvements in EDNO bioavailability are a major research focus to delineate novel drug targets aimed at improving endothelial function and cardiovascular disease (CVD) outcomes [15]. 

## 3. Diabetes-Mediated Endothelial Dysfunction

Extensive clinical evidence has demonstrated that diabetic patients have attenuated EDNO-dependent vascular tone [16, 17]. The resultant endothelial dysfunction is an important precursor of diabetes-mediated vascular events and has emerged as an independent risk factor for diabetes-associated cardiovascular complications [18]. 

**Figure 2:**
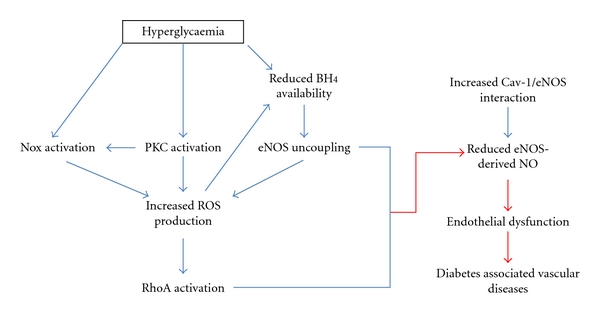
Pathways activated during hyperglycaemia that limit eNOS function and subsequent NO production, leading to endothelial dysfunction and diabetes-associated vascular disease.

Diabetic vessels from murine models and various endothelial derived cells from vascular beds stimulated with high glucose, exhibit increased levels of ROS associated with attenuated EDNO levels [19–21] (Figure 2). It is now widely accepted that in the diabetic milieu, through upregulation of the NADPH oxidase (Nox) enzymes [20, 22], ROS such as superoxide are released in pathological amounts and contribute to the observed reductions in EDNO bioavailability [23]. In particular, superoxide rapidly inactivates EDNO forming deleterious peroxynitrite, which in turn oxidizes the essential eNOS co-factor, BH_4_, thereby uncoupling the eNOS enzyme. Consequently, eNOS uncoupling diminishes the capacity of the enzyme to produce EDNO and causes a switch in production to superoxide, thereby further increasing superoxide levels [20]. The “eNOS uncoupling” phenomenon has gained an increasing amount of attention and is considered as one of the major sources of ROS production. Recently, it has been elucidated that in a prooxidative environment, eNOS is subjected to S-glutathionylation, an oxidative post-translational modification [24]. This modification occurs specifically at the cysteine 908 residue, thereby affecting the redox function of the enzyme and leading to an increase in superoxide production [24, 25]. Furthermore, eNOS S-glutathionylation is associated with impaired endothelium-dependent relaxation that is restored by thiol-specific reducing agents, which reverse the S-glutathionylation process, in hypertensive vessels [24]. 

The enhanced superoxide generating activity of the Nox family of enzymes plays a key role in mediating oxidative stress and endothelial dysfunction. In the vasculature, the predominant isoforms of the multisubunit Nox enzyme are the Nox1, Nox2, and Nox4 isoforms and their regulatory subunits, including p22phox, p47phox, and Rac-1 [26]. In streptozotocin- (STZ-) induced murine models and db/db mice, which represent T1D and T2D respectively, it has been shown that the expression and activity of these Nox isoforms and their regulatory subunits are greatly upregulated in the aortic region and mesenteric vascular bed [22, 27–29]. Furthermore, this upregulation was associated with increased oxidative stress and attenuated eNOS expression and eNOS-derived EDNO [29, 30]. More recently, it was shown that the increased expression of the Nox catalytic subunits, in particular Nox2, is directly related to eNOS uncoupling and eNOS-derived superoxide production in carotid arteries of diabetic rats [31]. 

Another cellular signaling pathway that is altered in the presence of high glucose and contributes to endothelial dysfunction is the increased de novo synthesis of diacylglycerides (DAG) and the subsequent activation of protein kinase C (PKC) [32, 33]. Indeed, various vascular cells exposed to hyperglycaemic conditions, as well as tissues from diabetic animals, have shown a consistent activation of PKC, which in turn modulates ROS generation and is described extensively in the review by Yang et al. [33–35]. Finally, recent studies have shed light on the pivotal role of the RhoA/Rock pathway (to be discussed in more detail below), which plays a part in diabetes-associated endothelial dysfunction via modulation of eNOS and EDNO levels [21].

Collectively, these studies show that various pathways are altered in diabetes and that these alterations influence the balance between ROS and EDNO production. It is becoming increasingly clear that this balance between ROS and EDNO (Figure 2) is critical for vascular health, function and integrity in diabetes. Therefore, a major research focus of the past decade has been towards the improvement of vascular endothelial function via modulation of these pathways in order to limit or prevent vascular complications associated with diabetes.

## 4. Improving Endothelial Function: A Clinical Perspective

Several therapeutic interventions have been tested in patients with diabetes and cardiovascular disease in an effort to improve endothelial function. These therapeutic interventions, in particular statin therapy, ACE inhibition, and arginine supplementation, have shown some improvements in these disease settings. Statins, which were initially designed as lipid-lowering drugs, demonstrate lipid-independent effects such as partially restoring EDNO levels and decreasing oxidative stress in hypertensive and hypercholesterolaemic patients [48, 49]. These improvements occurred more rapidly than the decline in cholesterol levels [48], suggesting that one of the pleiotropic effects of statins is to enhance endothelial function by upregulating EDNO signaling. Furthermore, long-term ACE inhibition improved forearm blood flow, a marker of improved vascular function, in patients with coronary artery disease and T2D in response to acetylcholine [50, 51]. Indeed, it was shown that ACE inhibition attenuated the superoxide-generating effects of angiotensin II, impaired the breakdown of bradykinin, and increased the production of EDNO in patients with coronary artery disease [50]. In addition, L-arginine supplementation was able to restore diabetes-mediated endothelial-dependent vasodilation by augmenting cGMP production in diabetic settings where L-arginine stores were depleted [52]. Although these therapeutic strategies are showing promise in restoring vascular function in diabetic patients, it is likely that a more targeted approach focusing specifically on the mechanisms that contribute to diabetes-mediated endothelial dysfunction will ultimately yield more promising outcomes. This review will now focus on the mechanisms and novel compounds that specifically target eNOS signaling and the regulation of oxidative stress in the context of a diabetic setting. 

## 5. Targeting eNOS Signaling and EDNO Production

eNOS is under constant regulation by various factors, including phosphorylation, transcriptional regulation, direct interaction with proteins, and substrate and co-factor availability. Furthermore, downstream effectors of various cellular signaling pathways, such as RhoA, are also capable of modulating eNOS function. In this section, we highlight the importance of BH_4_ availability, transcriptional regulation, and the role of RhoA and Cav-1 in proper eNOS regulation and signaling in a diabetic context (see Table 1). 

### 5.1. BH_4_ Availability

BH_4_ is a critical co-factor of eNOS regulation, facilitating electron transfer from its reductase domain to its oxygenase domain. For eNOS to be catalytically active, it must exist in its dimeric form. Indeed, BH_4_ contributes significantly to the stability of the eNOS dimer [39]. Several reports have indicated that hyperglycaemia results in significant reductions in BH_4_ levels, thereby “uncoupling” eNOS to its monomeric form and causing an increase in eNOS-derived superoxide [39, 40]. Furthermore, the diabetes-mediated increases in ROS levels, particular peroxynitrite, oxidizes BH_4_ to its inactive form dihydrobiopterin (BH2) [36]. A recent study has shown that BH_4_ oxidation is the key determinant for eNOS uncoupling and under conditions of low BH_4_ bioavailability, eNOS uncoupling is suppressed through increased association of eNOS with Cav-1 [53]. 

A clinical study has shown that concomitant intra-arterial infusion of BH_4_ in type 2 diabetic patients improved endothelium-dependent vasodilation, demonstrating the therapeutic potential of upregulating BH_4_ bioavailability [37]. Furthermore, BH_4_ supplementation improved endothelial function in vessels from animal models of hypercholesterolaemia and diabetes [36, 38]. The importance of BH_4_ availability was further strengthened by studies overexpressing the rate limiting enzyme guanosine triphosphate-cyclohydrolase 1 (GTPCH) which is involved in de novo synthesis of BH_4_ [39]. Adenoviral-mediated gene transfer of GTPCH in human endothelial cells exposed to high glucose conditions rescued eNOS function by increasing EDNO production and reducing superoxide levels as well as improving the stability of the eNOS dimer [39]. These results were corroborated in an *in vivo* transgenic GTPCH overexpressing mouse model of T2D, which exhibited markedly improved EDNO-dependent vascular tone, attenuated oxidative stress, and increased eNOS dimer to monomer ratio [40]. Thus, based on preclinical research and limited clinical data, augmentation of BH_4_ levels in diabetic patients, appears to be a feasible strategy to restore impaired endothelial dysfunction.

### 5.2. Transcriptional Regulation of eNOS

Various physiological and pathophysiological stimuli are able to modify the transcriptional activity of the eNOS gene by inducing transcription or stabilizing steady-state eNOS mRNA levels [54]. Increased transcription of the enzyme in turn results in sustained activation of eNOS-dependent activities. For instance, sheer stress has been implicated in modulating eNOS mRNA stability while growth factors, such as vascular endothelial growth factor (VEGF), stimulate transcription of the eNOS gene [54]. Chemical library screening for compounds that stimulate eNOS transcription yielded two small molecular weight compounds, named AVE9488 and AVE3085, which have demonstrated the ability to increase EDNO production while concurrently up-regulating eNOS gene expression and reversal of eNOS uncoupling [41, 43]. In apolipoprotein-E- (ApoE-) deficient mice, AVE9488 and AVE3085 reduced cuff-induced neotima formation and atherosclerotic plaques, while atherosclerotic plaque formation was unaffected in ApoE/eNOS double knockout (KO) mice, indicating that the actions of these compounds are eNOS-specific [41]. Furthermore, AVE3085 improved endothelial-dependent vascular function and lowered blood pressure in spontaneously hypertensive rats [43], and AVE9488 exhibited cardioprotective effects against ischemia-reperfusion injury in an EDNO-dependent manner [42]. Although, these transcriptional regulatory compounds have not been tested directly in a diabetic context, their ability to ameliorate endothelial dysfunction in pathophysiological settings, such as hypertension and atherosclerosis, lends support for their therapeutic potential in diabetes-induced endothelial dysfunction.

### 5.3. Potential Role of RhoA

RhoA is a small GTPase protein involved in several aspects of cellular function including signal transduction cascades related to vascular inflammation. Amongst its many regulatory functions, activation of RhoA and its downstream target, Rho-associated kinase (ROCK), has been shown to downregulate eNOS gene expression by affecting eNOS mRNA stability and suppressing protein kinase B/Akt activation, thus reducing eNOS phosphorylation and catalytic activity [12]. Conversely, administration of the ROCK inhibitor, fasudil, increased protein kinase B/Akt activity and EDNO release in cultured endothelial cells [44]. Additionally, fasudil administration was protective against vascular-injury-induced leukocyte recruitment in wild type but not eNOS KO mice [44], confirming that one of the targets of this ROCK inhibitor is downstream eNOS-dependent activites. Importantly, from a diabetes perspective, studies have demonstrated a significant correlation between increased RhoA activity and impaired vascular function in experimental models of T1D and T2D [21, 55, 56]. Recently, it was shown that increased levels of ROS, in particular peroxynitrite, suppress eNOS activity in a RhoA/ROCK-dependent manner in STZ-induced diabetic rats [12, 21, 55, 56]. Furthermore, treatment with the peroxynitrite decomposition catalyst, FeTTPs, improved vasorelaxation to acetylcholine, lowered oxidative-stress and RhoA activity, upregulated eNOS expression, and improved EDNO levels [21]. It has also been shown that the RhoA/ROCK pathway is involved in the pathogenesis of diabetic retinal microvasculopathy by promoting leukocyte and neutrophil adhesion to the retinal vasculature, thereby contributing to endothelial damage [57]. 

Inhibitors of the RhoA/ROCK pathway are showing promise as potential regulators of vascular damage. Non-isoform specific ROCK inhibitors such as fasudil and Y-27632 protect against various cardiovascular diseases such as atherosclerosis, pulmonary and systemic hypertension and chronic heart failure in clinical and preclinical studies [45–47]. Mechanistically, Y-27632 has been able to prevent thrombin-mediated downregulation of eNOS gene expression in cultured endothelial cells [58]. More importantly, fasudil has a direct effect on endothelial function, as demonstrated by improved vascular resistance and forearm blood flow during intra-arterial infusion of the ROCK inhibitor [46]. In addition, intravitreal administration of fasudil significantly increased eNOS activation and decreased leukocyte adhesion in the retinas of diabetic rats [57]. Thus, the diverse range of benefits associated with inhibiting the RhoA/ROCK pathway have paved the way for the generation of newer ROCK inhibitors with higher specificity between ROCK isoforms [59]. Currently, only fasudil is approved for human use in the treatment of acute ischemic stroke [60]. The testing of fasudil and newer more specific second generation ROCK inhibitors in a diabetic setting would be of great interest in an effort to limit vascular complications.

### 5.4. Modulating eNOS with Caveolin-1

Cav-1 is the main structural protein of endothelial cell caveolae, which are highly dynamic invaginations of the plasma membrane known to play an integral role in cellular signal transduction events [14]. Various endothelial cell signaling molecules, including eNOS, localize in caveolae and are modulated by direct interaction with Cav-1 [61]. One of the most intriguing roles of endothelial Cav-1 is its negative regulation of eNOS [61, 62]. Cav-1 has been shown to directly bind eNOS and to inhibit eNOS-derived EDNO release under basal conditions (Figure 1) [63]. On the contrary, for optimal EDNO release, eNOS must reside in caveolae microdomains. This was clearly demonstrated by a reduction in EDNO release from HEK 293 cells stably transfected with palmitoylation-deficient mutants of eNOS, which lacked the ability of eNOS to target caveolae [64]. Therefore, eNOS is most catalytically active when present in caveolae microdomains and dissociated from the Cav-1 protein. Accumulating evidence now suggests that in cardiovascular disease, endothelial dysfunction occurs as a result of alterations in the caveolae/Cav-1 signaling pathway [15]. Supportive evidence for a role for Cav-1 in proper vascular regulation comes from studies involving Cav-1 KO animals, which demonstrate dysregulated eNOS synthesis, increased vascular permeability, cardiomyopathy, and pulmonary hypertension [65, 66], all of which are rescued upon reintroduction of Cav-1 back into the endothelium [66, 67].

From the above studies, it is clear that for optimal eNOS activity, the presence of dissociated eNOS from Cav-1 in functional caveolae is required. However, both reductions in Cav-1 as well as overexpression of Cav-1 have been shown to affect eNOS activity in diabetic settings. For example, in a study of moderately diabetic rats, kidney cortical eNOS dimer-to-monomer ratios and eNOS phosphorylation were reduced, thereby contributing to the attenuation of proper EDNO synthesis. Interestingly, eNOS colocalized with Cav-1 throughout the renal vascular endothelium, however, the amount of Cav-1 bound to the plasma membrane was significantly attenuated [68]. These data clearly suggest that the dynamics of membrane bound eNOS/Cav-1 has to be preserved for proper EDNO synthesis. More recently, it has been shown that impaired endothelium-dependent vasorelaxation is linked to increased Cav-1 protein expression in the aorta of diabetic rats. This was attributed to an inhibition of eNOS function due to Cav-1 binding and a reduction in EDNO production [68, 69]. Furthermore, in a diabetic setting, the interaction of Cav-1 with other pathways has also been invoked. For example, Cav-1 has been implicated in diabetic peripheral neuropathy through regulation of neural cell growth factor receptor Erb 2 signaling [70].

Although a definite role for Cav-1 in diabetes has not been fully elucidated, it is apparent that modulating eNOS function by Cav-1 could be beneficial in ameliorating diabetes mediated endothelial dysfunction. A major issue that has limited Cav-1 research to date is that genetic ablation of Cav-1 results in the loss of both caveolae and Cav-1 dependent signaling [65]. This makes segregation of the functional role of caveolae versus Cav-1 dependent signaling pathways problematic. Hence, a novel approach to target the eNOS/Cav-1 interaction in a regulated fashion is clearly warranted. Previous studies have shown that Cav-1 binding to and inhibition of eNOS is mediated by the putative Cav-1 scaffolding domain (amino acids 82–101) [61]. By performing alanine scanning of the Cav-1 scaffolding domain, we have determined the amino acids responsible for eNOS inhibition [63]. Mutation of the amino acids threonine 90, 91 (T90, T91) and in particular phenylalanine 92 (F92) to an alanine, failed not only to inhibit eNOS activity but also was able to increase eNOS-derived NO release. This occurred despite Cav-1 retaining the ability to bind to eNOS [63, 71]. Moreover, we have generated a cell-permeable Cav-1 peptide lacking the eNOS inhibitory domain. When this peptide was applied *ex vivo* to aortic rings isolated from wild-type mice, there was an 80% improvement in endothelial-dependent vasodilation as compared to the wild-type peptide, an effect that was abolished in eNOS KO mice. This clearly indicates that eNOS is a specific target for the mechanism of action of this peptide. Furthermore, blood pressure was reduced *in vivo* following an intraperitoneal injection of the peptide in a concentration dependent manner. Lastly, in endothelial cells, treatment with the peptide was able to decrease superoxide production as measured by the cytochrome c assay [71]. Thus, our group has established a more targeted approach to positively regulate eNOS function by Cav-1. This strategy has the potential to improve endothelial dysfunction by upregulating eNOS activity in diabetes and cardiovascular disease, both of which have impaired Cav-1/eNOS signaling. However, a limitation that must be taken into account, is that increasing EDNO levels in the presence of oxidative stress, could lead to the production of peroxynitrite and consequently nitrosylation of proteins, leading to further damage.

## 6. Targeting Oxidant and Antioxidant Pathways in Diabetes

The vascular system controls excess ROS through a diverse range of endogenously expressed antioxidant enzymes, thus limiting oxidative damage. Antioxidants serve to protect against oxidative damage by scavenging ROS and interfering with downstream signaling events mediated by ROS. However, in diabetes, the activity of vascular ROS-producing enzymes is increased, whilst antioxidant defences are altered or impaired, skewing the balance to a more prooxidative profile. In this section, we discuss a major ROS producing enzyme of the vasculature and two key vascular antioxidant enzymes known to play a role in the protection against endothelial dysfunction and cardiovascular disease induced by diabetes. 

### 6.1. Nox Isoforms

Since the Nox family of enzymes is one of the primary sources of ROS in the vasculature, much interest has focused on ways to minimise ROS production without compromising the important role played by physiologically relevant concentrations of ROS [72]. Compounds that suppress Nox activity may therefore offer therapeutic benefits to ameliorate diabetic complications, in particular diabetes mediated endothelial dysfunction and atherosclerosis where Nox involvement is increasingly being appreciated [73]. Indeed, it has been suggested that inhibition of vascular smooth muscle-specific Nox1 may be an efficient strategy to suppress neointimal formation in the prevention of vascular complications associated with diabetes through the prevention of smooth muscle cell migration, proliferation and extracellular matrix production [74].

Several novel small-molecule and peptide inhibitors of the NOX enzymes have been developed and shown to have promise in experimental studies [75]. Indeed, treatment with the Nox inhibitor, apocynin, reversed the upregulation of Nox isoforms, increased eNOS function, and reduced endothelial dysfunction in STZ-induced and fructose-fed rats [29, 30]. Apocynin was also effective in improving vascular function induced by hyperglycaemia in a non-obese model of T2D [76]. Another novel Nox inhibitor, VAS2870, lowered superoxide formation in oxidized low-density-lipoprotein-treated endothelial cells [77]. A highly-specific Nox inhibitor, known as gp91 ds-tat, has been shown to interfere with Nox subunit assembly and subsequent activation of the enzyme. Although gp91 ds-tat has not been tested in a diabetes-specific setting, it has shown promise in a rat model of hypertension through its ability to decrease vascular ROS and endothelial dysfunction [78, 79].

Even though studies using Nox inhibitors have shown benefit, the issues of specificity, potency, and toxicity militate against any of the existing published compounds as candidates for drug development. For example, the mode of action of apocynin has been controversial and a matter of debate. Recent evidence has suggested that apocynin is nonselective in its mode of action as it also targets other enzymes such as Rho-kinase. Indeed, it is now believed to act as an antioxidant rather than a specific Nox inhibitor, as demonstrated in vascular endothelial and smooth muscle cells [80]. Thus, a more preferable strategy in the design of Nox-inhibitors would be the development of agents with isoform and target specificity. This strategy may be more beneficial considering the important signaling role provided by some of the Nox isoforms and the important protective role of Nox2 in the innate immune response.

### 6.2. Superoxide Dismutase

**Figure 3:**
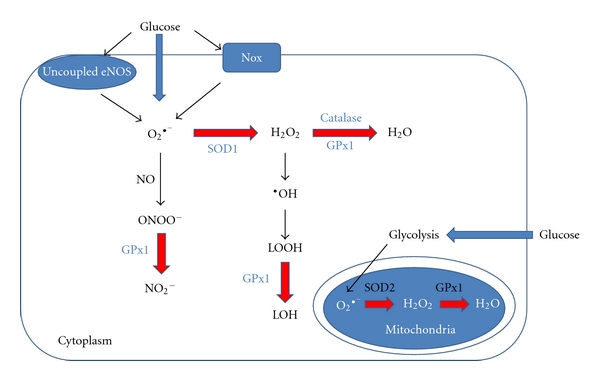
Classic antioxidant pathways for the neutralization of ROS produced by hyperglycaemia in the cytoplasm and mitochondria of endothelial cells. O_2_^•−^: superoxide anion; ONOO^−^: peroxynitrite; NO_2_^−^: nitrite; H_2_O_2_: hydrogen peroxide; ^•^OH: hydroxyl radicals; LOOH: lipid hydroperoxides; LOH: lipid alcohol; Nox: NADPH oxidase.

The superoxide dismutase (SOD) enzymes are involved in the removal of superoxide through its dismutation to oxygen and hydrogen peroxide. The SOD enzymes are therefore the first line of defence against increases in superoxide and for that reason are important regulators of ROS production (Figure 3) [81]. Of relevance to the vasculature, the SOD enzymes are also a key determinant of EDNO bioavailability since SOD competes with EDNO for superoxide in a diffusion-limited manner, thus attenuating the formation of peroxynitrite. In doing so, the SOD enzymes indirectly improve EDNO bioavailability [82]. Indeed, in mice with a genetic ablation of the cytosolic Cu/Zn-containing isoform, SOD1, endothelial dysfunction was associated with increased superoxide and peroxynitrite levels compared with wild type controls [83]. Furthermore, exogenously added SOD was able to partially restore endothelium-dependent vasodilation in an eNOS-dependent manner in SOD1 KO mice [83]. In addition, overexpressing the mitochondrial isoform, SOD2, specifically in the endothelium of STZ-induced diabetic mice, prevented diabetic retinopathy and superoxide-mediated oxidative stress [84]. These data clearly demonstrate the important role SOD plays in balancing oxidative stress through removal of superoxide and thereby maintaining EDNO levels.

Studies assessing the level of antioxidant defence in diabetes are mostly in agreement that antioxidant capacity is compromised in the diabetic milieu. Indeed, lower SOD1 activity has been associated with both T1D and T2D patients [85–87] and with increased susceptibility to vascular disease in children with T1D [88]. However, paradoxically in one study of a rabbit model of diabetes, impaired vascular function was linked to increased SOD expression and augmented superoxide levels [89]. However, adenoviral *ex vivo* gene transfer of both SOD1 and SOD2 into these diabetic rabbits improved vascular function by decreasing superoxide levels, leading these authors to conclude that, despite the increase in endogenous SOD, it had been rendered functionally less active and therefore unable to cope with the elevated diabetes-mediated oxidative stress [89]. This was confirmed in human endothelial cells, where exposure to high glucose for 7 and 14 days increased SOD1 and SOD2 protein levels despite SOD activity declining, therefore stressing the need for functionally active SOD to protect against oxidative stress [90].

Cardiovascular clinical trials investigating the potential of exogenous antioxidants, such as vitamin C and E, to bolster antioxidant defences have failed to show a positive outcome with respect to CVD endpoints [91, 92]. This lack of improvement in cardiovascular outcomes has spawned the development of compounds that mimic endogenous antioxidant enzymes with greater cellular penetration, efficacy, and stability, in order to lower oxidative stress in disease settings. Indeed, administration of tempol, a cell-permeable SOD mimetic, has shown improvements in diabetes associated microvascular complications, such as nephropathy and retinopathy [93, 94]. In addition, tempol restored endothelial vasorelaxation in large conduit vessels of alloxan-induced diabetic rabbits [95]. MN40403, another highly specific nonpeptide SOD mimetic was able to reverse endothelial dysfunction *ex vivo* by targeting Nox-mediated superoxide production in aortae of ApoE-deficient mice [96].

Lastly, it is important to note that although SOD and SOD mimetics have displayed efficacy in experimental models of diabetes, their role in improving diabetic vascular complications remains controversial. This may be due to the fact that in pathological conditions, dismutation of superoxide by SOD is linked to excess production of hydrogen peroxide, another ROS known to cause irreversible endothelial damage and attenuate EDNO production [97]. Thus, the availability or concurrent addition of other antioxidants such as catalase, thioredoxins, peroxiredoxins, and glutathione peroxidases, which function to neutralize hydrogen peroxide and/or hydroxyl radicals, may prove to be a more effective therapy to combat oxidative stress [81].

### 6.3. GPx1: A Versatile Antioxidant Enzyme

Glutathione peroxidases (GPx) are a family of selenocysteine-containing enzymes that participate in the second step of the antioxidant pathway, which involves the neutralization of hydrogen peroxide to water (Figure 3) utilizing glutathione (GSH) as its substrate [81, 98]. GPx1 is the predominant isoform expressed in the cytosol and mitochondria. Under conditions of increased oxidative stress, a build-up of hydrogen peroxide favours the production of hydroxyl radicals through Fenton-type reactions, leading to the formation of lipid peroxides. The formation of lipid peroxides in turn damage cell membranes and contribute to the pathogenesis of atherosclerosis [99]. GPx1 is considered a multifaceted antioxidant enzyme in its ability to eliminate lipid peroxides, hydrogen peroxide [81] and reduce the potent peroxynitrite anion [81, 100] (Figure 3). Indeed, recent preclinical and clinical data have shown that GPx1 plays a critical role in protecting the cardiovascular system against oxidative stress [101, 102] with low levels of GPx1 activity acknowledged as an independent risk factor for cardiovascular events in patients with coronary artery disease [102].

The generation of GPx1 KO mice by our group [103] and others [104, 105] have specifically demonstrated the importance of GPx1 in protecting the vasculature against oxidative stress and vascular complications of diabetes [106]. Although deletion of GPx1 is nonlethal and GPx1 KO mice are fertile [81, 103], these mice have an enhanced oxidative stress profile and are more susceptible to endothelial dysfunction [107, 108]. Indeed, a 50% reduction of GPx1, as seen in heterozygous mice, is sufficient to impair endothelial function and cause significant abnormalities to the vasculature and cardiac structures [101, 108]. Furthermore, resistance arteries isolated from GPx1 KO mice displayed paradoxical vasoconstriction in response to bradykinin, a EDNO-dependent vasodilator, whilst their wild-type counterparts exhibited dose-dependent vasodilation [107]. These findings were accompanied by reduced levels of cGMP, a downstream signaling molecule of the EDNO pathway, with no change in eNOS expression within the aorta of GPx1 KO mice [107]. These findings are strongly suggestive that a deficiency in GPx1 contributes to the reduction in EDNO bioavailability. In support of this finding, Galasso et al. demonstrated impaired ischemia-induced angiogenesis, as well as a reduction in the number of endothelial progenitor cells in response to vascular endothelial growth factor stimulation in GPx1 KO mice, both processes known to be EDNO dependent [109]. In addition, GPx1 deficiency has been shown to accelerate angiotensin-II-mediated endothelial dysfunction, cardiac hypertrophy, and mean arterial blood pressure [110, 111].

Armed with this knowledge, our group was particularly interested in the role of Gpx1 in vascular complications associated with diabetes. In order to achieve these aims, we generated ApoE/GPx1 double KO (dKO) mice and rendered them diabetic using STZ, which proved to be a robust model for diabetes-associated atherosclerosis and nephropathy [106, 112]. In diabetic ApoE/GPx1 dKO mice, atherosclerotic lesions were significantly increased in all regions of the aorta and aortic sinuses compared with diabetic ApoE KO mice. The enhanced atherosclerosis was accompanied by an increase in proatherogenic inflammatory markers, such as VCAM-1 and Nox2, and the pro-fibrotic markers, CTGF and VEGF [106]. In the absence of GPx1, there was an upregulation in SOD 1 and catalase, however, this compensatory response was insufficient to protect the mice against oxidative stress-mediated damage, as quantified by the increased nitrotyrosine levels detected in ApoE/GPx1 dKO compared with ApoE KO counterparts [106]. Diabetic nephropathy, detected as increased albuminuria, creatinine clearance, mesangial expansion, oxidative stress, and fibrosis, was also significantly enhanced in diabetic ApoE/GPx1 dKO mice compared with diabetic ApoE KO controls [112]. Collectively, these studies implicate a major role for GPx1 in protecting the vasculature against hyperglycaemia-mediated oxidative stress, endothelial dysfunction, atherogenesis, and nephropathy.

Ebselen is a lipid-soluble seleno-organic compound, which mimics the activity of GPx1. Clinical studies have demonstrated that ebselen has neuroprotective effects in stroke patients, showing its suitability and safety for human usage. In addition, Ebselen has been extensively studied for its therapeutic potential in various experimental models of diabetes [112–115]. In a T2D rat model associated with metabolic syndrome, a reduction in endothelium-dependent vasodilation as well as EDNO production and angiogenic capacity was prevented by treatment with ebselen [115]. Additionally, we have shown that ebselen is protective against atherosclerosis in diabetic ApoE KO mice, which was associated with a decrease in oxidative stress markers and reduced expression of proatherogenic cellularity and mediators [113]. In endothelial cells, pretreatment with ebselen, diminished hydrogen-peroxide induced increases in inflammatory cytokines, such as TNF-*α* and NFkB, and attenuated the TNF-*α*-induced expression of endothelial cell adhesion molecules (VCAM-1 and ICAM-1) [113, 116]. Importantly, in our studies, ebselen was able to significantly improve diabetes-associated atherosclerosis and nephropathy in our ApoE/GPx1 dKO mice [112]. This finding was pivotal as it demonstrated that ebselen, a synthetic compound that mimics an endogenous antioxidant enzyme, is target specific and able to lessen the pathological consequences brought about by a deficiency in GPx1.

Currently, newer ebselen analogues with structural modifications, including a diselenide moiety, have been generated for their greater efficacy, potency, and their potential to behave in a superior fashion to ebselen *in vivo*. Indeed, thorough biochemical and kinetic analysis have proven that these analogues have a higher catalytic activity [117]. Most recently, Hort et al. have shown that daily treatment of hypercholesterolemic LDL receptor KO mice with diphenyl diselenide (0.1 mg/kg or 1 mg/kg) for 30 days, reduced atherosclerotic lesions, which was accompanied by improved vascular function, downregulation of proatherogenic genes, reduced infiltration of inflammatory cells, and lowered oxidative stress levels [118]. These findings are supported by our own unpublished observations with various other ebselen analogues designed for their improved activity, which have demonstrated their antiatherogenic potential in diabetic ApoE/GPx1 dKO mice. Accordingly, targeting GPx1 activity through the use of novel GPx1 mimetics is proving beneficial in various preclinical settings, providing a strong foundation for their translation into clinical trials.

## 7. Conclusion

It is now well accepted that proper regulation of endothelial function in diabetes is considerably impaired, leading to often fatal cardiovascular complications. EDNO bioavailability and oxidative stress are two separate but inter-linked determinants of vascular function that are both compromised in a diabetic setting. Hence, developing novel therapeutics to specifically improve EDNO bioavailability and reduce oxidative stress is a clearly desirable dual strategy to manage endothelial dysfunction. This review highlights the importance of developing new compounds that upregulate EDNO synthesis, target key vascular ROS-producing enzymes, and mimic endogenous antioxidants, a strategy that might prove clinically relevant in preventing the development and/or retarding the progression of diabetes associated vascular complications.

## Figures and Tables

**Table 1:** Novel targets that regulate eNOS function.

Compound	Biological action	Disease context	References
BH_4_	↑co-factor availability	Endothelium-dependent vasodilation, hypercholesterolemia, diabetes	[36–38]
GTPCH	↑BH_4_ synthesis	Diabetes	[39, 40]
AVE9488	↑eNOS transcription	Atherosclerosis, ischemia-reperfusion injury	[41, 42]
AVE3085	↑eNOS transcription	Atherosclerosis, hypertension	[41, 43]
Fasudil	Stabilise eNOS gene expression, ↑eNOS activity	Atherosclerosis, hypertension, heart failure, diabetes	[44–47]
